# Placental malaria among HIV-infected and uninfected women receiving anti-folates in a high transmission area of Uganda

**DOI:** 10.1186/1475-2875-8-254

**Published:** 2009-11-14

**Authors:** Patrick M Newman, Humphrey Wanzira, Gabriel Tumwine, Emmanuel Arinaitwe, Sarah Waldman, Jane Achan, Diane Havlir, Philip J Rosenthal, Grant Dorsey, Tamara D Clark, Deborah Cohan

**Affiliations:** 1 School of Medicine, University of California, San Francisco, 513 Parnassus Ave, San Francisco, CA 94131, USA; 2 Infectious Diseases Research Collaboration, PO Box 7475, Kampala, Uganda; 3 Department of Molecular Biology and Biotechnology, Makerere University, PO Box 7062, Kampala, Uganda; 4 Department of Pediatrics, College of Health Sciences, Makerere University, PO Box 7072, Kampala, Uganda; 5 Department of Medicine, University of California, San Francisco, San Francisco General Hospital, 995 Potrero Ave, Ward 84, San Francisco, CA, 94110, USA; 6 Department of Obstetrics, Gynecology and Reproductive Sciences, University of California, San Francisco, San Francisco General Hospital, 1001 Potrero Ave, Ward 6D, San Francisco, CA 94110, USA

## Abstract

**Background:**

HIV infection increases the risk of placental malaria, which is associated with poor maternal and infant outcomes. Recommendations in Uganda are for HIV-infected pregnant women to receive daily trimethoprim-sulphamethoxazole (TS) and HIV-uninfected women to receive intermittent sulphadoxine-pyrimethamine (SP). TS decreases the risk of malaria in HIV-infected adults and children but has not been evaluated among pregnant women.

**Methods:**

This was a cross sectional study comparing the prevalence of placental malaria between HIV-infected women prescribed TS and HIV-uninfected women prescribed intermittent preventive therapy with sulphadoxine-pyrimethamine (IPT-SP) in a high malaria transmission area in Uganda. Placental blood was evaluated for malaria using smear and PCR.

**Results:**

Placentas were obtained from 150 HIV-infected women on TS and 336 HIV-uninfected women on IPT-SP. The proportion of HIV-infected and HIV-uninfected women with placental malaria was 19% vs. 26% for those positive by PCR and 6% vs. 9% for those positive by smear, respectively. Among all infants, smear+ placental malaria was most predictive of low birth weight (LBW). Primigravidae were at higher risk than multigravidae of having placental malaria among HIV-uninfected, but not HIV-infected, women. Adjusting for gravidity, age, and season at the time of delivery, HIV-infected women on TS were not at increased risk for placental malaria compared to HIV-uninfected women on IPT-SP, regardless of the definition used.

**Conclusion:**

Prevalence of placental malaria was similar in HIV-infected women on TS and HIV-uninfected women on IPT-SP. Nonetheless, while nearly all of the women in this study were prescribed anti-folates, the overall risk of placental malaria and LBW was unacceptably high. The population attributable risk of placental malaria on LBW was substantial, suggesting that future interventions that further diminish the risk of placental malaria may have a considerable impact on the burden of LBW in this population.

## Background

Infection with *Plasmodium falciparum *during pregnancy is associated with placental infection and a wide range of poor maternal, obstetrical and infant outcomes, including maternal anaemia, intrauterine growth restriction, preterm delivery, and low birth weight (LBW) [1]. Indeed, an estimated 100,000 infants die annually as a result of maternal *P. falciparum *infection [2]. Malaria and HIV represent synergistic epidemics in sub-Saharan Africa, and HIV-infected women are particularly vulnerable to the effects of malaria in pregnancy. HIV-infected pregnant women have significant alterations in both cellular and humoral immunity to malaria [3,4]. As a result, HIV-infected women, regardless of parity, experience worse sequelae from malarial infection and are at increased risk of malarial illness and placental malaria compared to HIV-uninfected women [5]. A study in Zimbabwe observed that dual infection with HIV and malaria was associated with a higher risk of maternal and infant mortality than infection with either HIV or malaria alone [6].

Since the mid 1990s, a standard practice in pregnancy in countries with stable malaria transmission has been intermittent preventive therapy (IPT) with two doses of sulphadoxine-pyrimethamine (SP) after quickening [7]. This practice has been demonstrated to reduce the risk of placental malaria, LBW and maternal anaemia [8-10]. IPT with SP is not indicated in HIV-infected pregnant women if they are already receiving daily trimethoprim-sulphamethoxazole (TS) for prevention of HIV-related complications, as SP and TS act by the same mechanisms and may be associated with overlapping toxicities [11]. TS is effective for malaria prevention in HIV-infected children and adults [12,13], but it has not been systematically evaluated among pregnant women. Data are urgently needed to address this question because many sub-Saharan countries, including Uganda, recommend daily TS for all HIV-infected pregnant women [5,14].

Complicating the use of TS and SP has been resistance in *P. falciparum *to anti-folate anti-malarial drugs, which arises from mutations in the dihydrofolate reductase (*dhfr*) and dihydropteroate synthetase (*dhps*) genes. Five mutations that are common in East Africa (*dhfr*-N51I, -C59R, and -S108N, and *dhps*-A437G and -K540E), and in particular *dhfr*-C59R and *dhps*-K540E, predict poor clinical response after treatment of children with malaria with SP [15,16]. Higher-level resistance to SP is conferred by the *dhfr *I164L mutation, which is rare in Africa, but has recently been seen in Uganda [13,17]. In vitro studies have shown cross-resistance between trimethoprim and pyrimethamine and between sulphamethoxazole and sulphadoxine [18]. The impact of these agents on drug resistance in placental malaria has not previously been reported.

This cross-sectional study compared placental malaria among HIV-infected women prescribed daily TS and HIV-uninfected women prescribed IPT with two doses of SP and presenting for delivery at a district hospital in Tororo, Uganda, a region of high HIV and malaria prevalence [19].

## Methods

### Study participants and data collection

**Figure 1:**
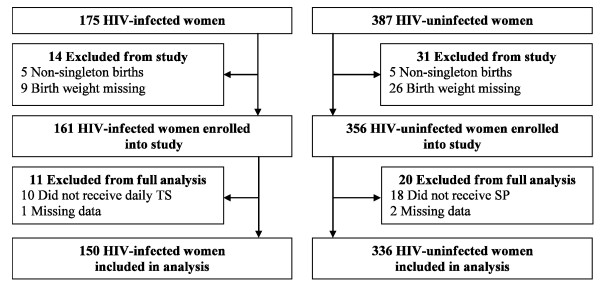
**Subject recruitment flowchart**. TS = trimethoprim-sulphamethoxazole; SP = sulphadoxine-pyrimethamine.

All HIV-infected women presenting to the Tororo District Hospital (TDH) Labor Ward for delivery between February 2008 and February 2009 were recruited. In addition, all HIV-uninfected women delivering between May 9 and May 23, 2008 and, subsequently, three consecutive HIV-uninfected women delivering after each delivery of an HIV-infected woman between July 2008 and February 2009 were included. [See Figure 1.] HIV status was known for all women delivering at TDH during the study period because of 100% uptake of antenatal HIV testing at this particular site.

Following delivery, study staff obtained demographic data, HIV status, data on receipt of TS or IPT-SP, data on receipt of antiretroviral therapy and obstetrical history from participants' antenatal cards and the birth log maintained on the Labor Ward. Gestational age at the time of delivery was estimated by Naegle's rule using self-reported last menstrual period recorded on the antenatal card at the first prenatal visit. Infants were weighed using a calibrated electronic scale within 24 hours of life. Women were categorized as primigravidae if the index pregnancy was their first; all other women were defined as multigravidae. Multigravidae were further stratified as gravida 2 (index pregnancy was their second) or gravida 3+ (index pregnancy was their third or more).

### Laboratory methods

Within 30 minutes of delivery, staff collected 2 mL of placental blood into sterile EDTA tubes via the incision method [20]. In brief, staff made a shallow incision into the maternal side of the placenta using sterile scissors and collected blood that pooled from the intervillous space. Within 24 hours of placental blood collection, staff prepared thick placental blood smears and filter paper specimens. Thick smears were air dried and stained for 30 minutes using 2% Giemsa. Experienced microscopists read the thick smears at 100× using immersion oil. Positive placental blood smear was defined as parasite density ≥ 1 parasite/ul. Smears were read as negative after review of 100 high-powered fields. Every slide was read by two different microscopists, blinded to each others' readings, and discrepant readings were settled by a third microscopist. Filter paper specimens were collected onto Whatman 5 M filter paper, and stored in individual sterile plastic envelopes containing desiccant. Parasite DNA was isolated from filter paper blood samples with chelex, as previously described [21], and samples were subjected in duplicate to species-specific nested PCR for *P. falciparum *[22]. Amplified DNA was run on 2.5% agarose gels containing ethidium bromide, and analyzed under UV light. Positive PCR of placental blood was defined as the presence of a species-specific band of amplified DNA.

Samples that showed *P. falciparum *by PCR also underwent analysis for polymorphisms in *dhfr *and *dhps *using nested polymerase chain reaction (PCR) followed by mutation-specific restriction enzyme digestion, as previously described [23]. Digestion products were visualized by electrophoresis and results classified as wild type or mutant; mixed results were classified as mutant.

Placental malaria was defined in three ways, all based on analysis of placental blood: 1) placental malaria detected by PCR (PCR+), 2) placental malaria detected by PCR but not by blood smear (PCR+/smear-), 3) placental malaria detected by blood smear (smear+).

### Statistical analysis

Demographic and birth outcome data on HIV-infected and HIV-uninfected women were compared using the chi-squared test for dichotomous variables, t-test for normally distributed continuous variables and Wilcoxon-Mann Whitney test for non-normally distributed variables. The association between the different definitions of placental malaria and LBW (birth weight < 2.50 kg) was measured using the chi-squared test or Fisher's exact test as appropriate. In particular, the magnitude of the association between LBW and PCR+/smear- versus smear+ placental malaria was compared to evaluate whether the addition of PCR to the definition of placental malaria was more predictive of LBW. The relationship between gravidity and risk of PCR+/smear- placental malaria was assessed using Fisher's exact test, stratified by HIV status. Population attributable risks were calculated to estimate the impact of placental malaria on the burden on low birth weight in the population [24]. In addition, the association between HIV status and molecular markers of anti-folate resistance was calculated. Lastly, HIV infection in the setting of daily TS was assessed as a risk factor for placental malaria with logistic regression models. All statistical analyses were calculated using StataSE version 10 (STATACorp LP, College Station, Texas).

### Ethical approval

Approval was obtained from the Ugandan National Council on Science and Technology and the Committee on Human Research at the University of California, San Francisco prior to launching this study.

## Results

### Patient characteristics

Demographic characteristics and birth outcomes of the 161 HIV-infected and 356 HIV-uninfected women are shown in Table 1. HIV-infected women were significantly older, less likely to be primigravidae and more likely to deliver prior to 37 weeks gestation as compared to HIV-uninfected women. Information was obtained on antiretroviral therapy prescribed for 94 (60%) of the 161 HIV-infected women - 14 (15%) received no antiretroviral therapy, 68 (62%) received abbreviated mono- or dual anti-retroviral therapy with zidovudine, stavudine, zidovudine/lamivudine or stavudine/lamivudine for the prevention of mother-to-child transmission of HIV (PMTCT), and 22 (23%) received nevirapine-based highly active antiretroviral therapy (HAART).

**Table 1:** Characteristics and birth outcomes of study participants.

Variable	HIV-infected mothers (n = 161)	HIV-uninfected mothers (n = 356)	p-value
Median maternal age in years (IQR)	28 (23-33)	23 (19-29)	< .001
Gravidity			
G1	31 (19)	134 (38)	< .001
G2	24 (15)	50 (14)	
G3 and above	106 (66)	172 (48)	
Chemoprevention during pregnancy			
IPT-SP	4 (2)	336 (94)	N/A
TS	143 (89)	0 (0)	
Both	7 (4)	0 (0)	
None	6 (4)	18 (5)	
Unknown	1 (1)	2 (1)	
Live birth	159 (99)	347 (97)	.35
Preterm delivery (< 37 weeks)	50 (31)	82 (23)	.05

Data are number (%) of patients, unless otherwise indicated; IQR = interquartile range; IPT-SP = intermittent preventive therapy with sulphadoxine-pyrimethamine; TS = daily trimethoprim-sulphamethoxazole; SD = standard deviation.

Overall, a high proportion (94%) of patients received the recommended anti-malarial prophylaxis (TS for HIV-infected and IPT-SP for HIV-uninfected women). The analyses that follow include only HIV-infected women prescribed TS and HIV-uninfected women prescribed IPT-SP.

### Relationship between placental malaria and low birth weight

Placental malaria detected by PCR was predictive of LBW among infants born to HIV-infected women on TS (RR 2.57; 95% CI 1.17-5.61) and HIV-uninfected women who received IPT (RR 2.18; 95% CI 1.26-3.75). [Data not shown.] PCR+/smear- placental malaria was predictive of low birth weight among HIV-uninfected women, though this was not observed among HIV-infected women (see Table 2). Placental malaria detected by blood smear was most predictive of low birth weight among infants born to HIV-infected and HIV-uninfected women.

**Table 2:** Association between placental malaria and low birth weight, stratified by maternal HIV status.

	Categories of placental malaria	N	Median birth weight (IQR)	LBW (%)	RR (95% CI)
Infants born to HIV- infected mothers on TS (n = 150)	No Infection	120	3.10 (2.70-3.40)	13 (11)	1.0 (reference)
	PCR +/Smear -	21	2.90 (2.60-3.20)^a^	3 (14)	1.32 (0.41-4.23)
	Smear +	9	2.30 (2.20-3.00)^b^	5 (56)	5.13 (2.36-11.16)

Infants born to HIV- uninfected mothers on IPT-SP (n = 336)	No Infection	249	3.10 (2.70-3.40)	25 (10)	1.0 (reference)
	PCR +/Smear -	57	2.80 (2.60-3.30)^c^	12 (21)	2.10 (1.12-3.92)
	Smear +	30	2.70 (2.50-3.00)^d^	7 (23)	2.32 (1.10-4.91)

IQR = interquartile range; LBW = Low birth weight (birth weight < 2.50 kg); RR = Relative risk; 95% CI = 95 Percent confidence interval; TS = daily trimethoprim-sulphamethoxazole; IPT-SP = intermittent preventive therapy with sulphadoxine-pyrimethamine; PCR+/Smear-: placental malaria detected by PCR, but not by blood smear, Smear +: placental malaria detected by blood smear; Comparisons of relative risks of groups with 5 or fewer cases of placental malaria use Fisher's exact test, other comparisons use the chi-squared test.

^a ^p-value = .12 compared to infants born to HIV-infected mothers with no infection

^b ^p-value < .01 compared to infants born to HIV-infected mothers with no infection

^c ^p-value < .05 compared to infants born to HIV-uninfected mothers with no infection

^d ^p-value < .01 compared to infants born to HIV-uninfected mothers with no infection

The population attributable risk (PAR) of smear+ placental malaria on LBW was 19% and 8% among HIV-infected and HIV-uninfected women, respectively. When the definition was expanded to include PCR+ placental malaria, the population attributable risk of placental malaria on LBW was 23% and 23% among HIV-infected and HIV-uninfected women, respectively.

### Relationship between HIV status and placental malaria

HIV-infected women on daily TS had a similar prevalence of placental malaria as compared to HIV-uninfected women on IPT-SP, regardless of the definition. In particular, the proportion of HIV-infected and HIV-uninfected women with placental malaria was 19% vs. 26% for PCR+, 14% vs. 17% for PCR+/smear- and 6% vs. 9% for smear+ placental malaria, respectively. The placenta from a single HIV-infected woman was negative by PCR but positive by smear.

**Figure 2:**
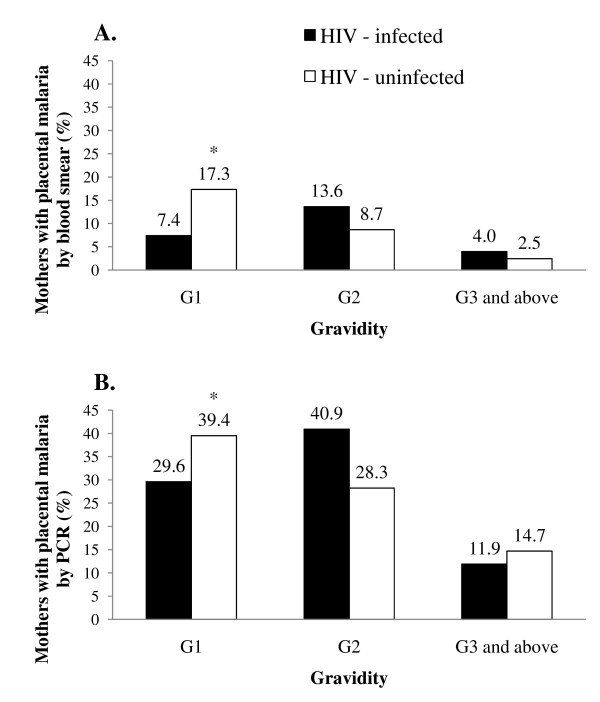
**Proportion of women having placental malaria stratified by HIV status**. A. = placental malaria detected by blood smear; B. = placental malaria detected by PCR (B). G1 = gravida 1; G2 = gravida 2; G3 and above = gravida 3 or more. * p < .01 for comparison of prevalence between primigravidae vs. multigravidae.

Among HIV-uninfected women on IPT-SP, primigravidae were at significantly higher risk of placental malaria as compared to multigravidae, whether defined by a positive blood smear (RR 4.53, 95% CI 2.08-9.86) or positive PCR (RR 2.22, 95% CI 1.55-3.20) (see Figure 2). Among HIV-infected women on daily TS, on the other hand, primigravidae were not at increased risk of placental malaria as compared to multigravidae. Moreover, there was no difference in the proportion of placental malaria that was PCR+/smear- among HIV-infected women at all gravidity (p-value = 1.0) (see Table 3). On the other hand, among HIV-uninfected women on IPT-SP, there was a trend towards a higher proportion of PCR+/smear- placental malaria among primigravidae compared to multigravidae (p-value = .07).

**Table 3:** Proportion of placenta malaria found to be PCR+/smear-, stratified by gravidity and maternal HIV status

Maternal HIV status	Gravidity	PCR+/smear-	Smear+	p-value
HIV-infected (n = 30)	G1	6 (75)	2 (25)	1.00
	G2	6 (67)	3 (33)	
	G3+	9 (69)	4 (31)	

HIV-uninfected (n = 87)	G1	28 (56)	22 (44)	.07
	G2	9 (69)	4 (31)	
	G3+	20 (83)	4 (17)	

Data are number (%) of patients; PCR+/smear -: placental malaria detected by PCR, but not by blood smear, Smear+: placental malaria detected by blood smear; P-value measuring the association between gravidity and PCR+/smear- placental malaria using Fisher's exact test.

Adjusting for gravidity, age and season at the time of delivery in a multivariable model, HIV-infected women on daily TS were not at increased risk for placental malaria as compared to HIV-uninfected women on IPT-SP whether defined by blood smear (adjusted odds ratio [OR] 1.04, 95% CI 0.45-2.38) or PCR (OR 0.89, 95% CI 0.53-1.49).

### Molecular markers of drug resistance

Molecular marker results were available for 130 (98%) of the 133 PCR+ placentas in the study, including 35 from HIV-infected and 95 from HIV-uninfected women. Of these 130 placentas, 43 were smear+, and 87 were PCR+/smear -. Considering three markers of resistance to TS and SP in placentas from this group, mutant genotypes for *dhfr-*59R and *dhps-*437G and -540E were observed in 32 (91%), 35 (100%), and 34 (97%) of HIV-infected women, and in 88 (93%), 93 (98%), and 92 (97%) of HIV-uninfected women, respectively. No significant difference in the prevalence of *dhfr-*59R (RR 0.99, 95% CI 0.88-1.11), *dhps-*437G (RR 1.02, 95% CI 0.99-1.05), or *dhps-*540E (RR 1.00, 95% CI 0.94-1.07) resistance markers was observed between HIV-infected and uninfected women. Mutant genotypes for *dhfr-*59R and *dhps-*437G and -540E were observed in 41 (95%), 42 (98%), and 42 (98%) of smear+ placentas, and in 79 (91%), 86 (99%), and 84 (97%) of PCR+/smear - placentas, respectively. No significant difference in the prevalence of dhfr-59R (RR 1.05, 95% CI 0.96-1.15), dhps-437G (RR 0.99, 95% CI 0.94-1.04), or dhps-540E (RR 1.01, 95% CI 0.95-1.08) resistance markers was observed between PCR+/smear - and smear+ placentas. The *dhfr*-164L mutant genotype, a marker of high-level SP resistance, was identified in only one, HIV-infected, woman. No mixed genotypes were observed for any marker.

## Discussion

This study found a high prevalence of placental malaria and low birth weight despite anti-folate use among women living in Tororo, a region of very high malaria transmission. Indeed, a very high proportion of women delivering at Tororo District Hospital received appropriate anti-malarial prophylaxis and, given the sampling technique, this is likely representative of women who experience hospital births in this region. As has been seen in other studies, a high proportion of LBW was attributable to placental malaria [1]. Among HIV-uninfected women, placental malaria was most prevalent among primigravidae, and multigravidae were at decreased risk, as has been seen in many prior studies [25-27]. This pattern was not observed in HIV-infected women, for whom gravidity did not significantly alter the risk of placental malaria. This difference in risk between HIV-infected and uninfected women has also been described previously [2,5,28].

The protective efficacy of daily TS against malaria among pregnant women has not been established. As daily TS is the standard of care for all those with HIV infection in Uganda, it is not possible to randomize HIV-infected pregnant women to daily TS versus IPT-SP. Rather, the prevalence of placental malaria was compared between HIV-uninfected women on IPT-SP and HIV-infected women on daily TS to estimate efficacy. The prevalence of placental malaria was similar between HIV-infected women on daily TS and HIV-uninfected women on IPT-SP. Numerous studies have demonstrated that HIV infection nearly doubles the risk of placental malaria [5,28,29]. Observational studies and trials suggest that monthly IPT-SP regimens can decrease this risk to levels seen among HIV-uninfected women receiving 2-dose IPT [30,31]. These data suggest that daily TS can, similarly, decrease the risk of placental malaria in the setting of HIV infection.

Placental malaria infection serves as the primary surrogate outcome for evaluating malaria in pregnancy because it is associated with adverse maternal and fetal outcomes [32]. One of the most important outcomes of placental malaria is LBW. The most common method of diagnosing placental malaria remains thick blood smear of placental blood, but many studies have used PCR of placental blood. Prior studies correlating PCR+ and blood smear+ placental malaria with LBW have produced conflicting results [33,34]. Although PCR is more sensitive than placental blood smear for malaria detection, the clinical predictive value of placental malaria diagnosed by PCR remains unclear, especially in the setting of HIV [35]. In this study, placental malaria determined by either PCR or smear of placental blood was associated with LBW. Further, these data suggest that placental malaria detected by blood smear may be the most clinically relevant definition. Specifically, a statistically significant association between PCR+/smear- placental malaria and LBW was found only among HIV-uninfected women, although this study was underpowered to detect such an association among HIV-infected women. Indeed, the underlying mechanism leading to PCR+/smear- placental malaria in this population receiving anti-folate prophylaxis remains unknown. PCR+/smear- placental malaria may be seen simply due to increased sensitivity of PCR compared to blood smear for identification of low-level infections. A more intriguing explanation is that PCR+/smear- placental malaria may represent past *P. falciparum *infection of the placenta with successful clearance of the parasite by either IPT-SP or daily TS. In this scenario, the placenta is exposed to active infection for a shorter period of time and, therefore, the harmful effects on the fetus are mitigated. If this explanation is true, the data here suggest that HIV-uninfected women on IPT-SP were more able to clear *P. falciparum *infection of the placenta as their gravidity increased. On the other hand, the HIV-infected multigravidae in this study were no more likely to clear infection than primigravidae, likely due to altered immune-recognition in the setting of HIV.

One concern regarding use of IPT or daily TS is the selection of drug-resistant infections. In this study, the prevalence of markers of anti-folate resistance did not differ between HIV-infected women on TS and HIV-uninfected women on SP, although baseline prevalence of these polymorphisms is very high in Tororo [36], limiting the ability to recognize selection of the polymorphisms by anti-folate therapy. These results suggest that the comparison of HIV-infected and uninfected women was not confounded by differences in resistance profiles in parasites infecting the two groups.

This study had several limitations. The comparable prevalence of placental malaria between HIV-infected and HIV-uninfected women may have been biased by unmeasured confounders including insecticide-treated bed net use or other protective factors associated with both risk of placental malaria and differential antenatal care between HIV-infected and HIV-uninfected women. Moreover, while available antenatal cards recorded provision of IPT and daily TS as appropriate based on HIV status, this study was unable to confirm compliance with this intervention. HIV-infected and HIV-uninfected groups were recruited at slightly different times of the year, potentially introducing sampling bias. Nonetheless, the holoendemic transmission of malaria in Tororo [37] and the long gestational period during which placental malaria can be acquired make this sampling bias unlikely. Similarly, logistic regression models including or excluding season at the time of delivery did not alter the findings. Although the study population was restricted to women delivering in the hospital, about two-thirds of Ugandan women overall give birth outside of health facilities [38], and 45% of HIV-infected women engaged in the CDC-run HIV treatment program in Tororo deliver at home. (Jaco Homsy, personal communication) As such, these findings may not be generalizable to those women delivering outside the hospital setting. Lastly, data on antiretroviral use were available for approximately 60% of the HIV-infected women in this study and, thus, this study could not systematically evaluate the association between immune reconstitution and risk of placental malaria.

Despite these limitations, this study provides important data on the epidemiology and clinical implications of placental malaria among HIV-infected women on daily TS delivering at a district hospital. As daily TS is increasingly becoming the standard of care for HIV-infected pregnant women in sub-Saharan Africa, these data offer a baseline against which to compare future anti-malarial interventions. Furthermore, these data suggest that placental malaria detected by blood smear is an appropriate surrogate outcome for future studies involving these populations. Larger studies investigating the clinical relevance of other measures of placental malaria among HIV-infected women on daily TS, including PCR and histopathology, are warranted. Given the time and resource-intensive nature of histopathology, it would particularly interesting to assess whether PCR+/smear- placental malaria correlates with past or chronic *P. falciparum *infection on histopathology in this population. Future studies should also include assessments of other sequelae of malaria in pregnancy, including maternal anaemia, late spontaneous abortion and infant mortality.

## Conclusion

In summary, the prevalence of placental malaria in Tororo was similar in HIV-infected women taking TS and HIV-uninfected women taking IPT. Nonetheless, while nearly all of the women in this study were prescribed anti-folates, the overall risk of placental malaria and LBW was unacceptably high. The population attributable risk of placental malaria on LBW was substantial, suggesting that future interventions that further diminish the risk of placental malaria may have a considerable impact on the burden of LBW in this population. In the setting of increased resistance of *P. falciparum *to anti-folate drugs, such interventions might include non-anti-folate based regimens [39,40]. Given the vulnerability of women and their infants to the detrimental effects of malaria and the potential to substantially diminish the risk of LBW and other adverse sequelae by reducing placental malaria, new strategic interventions merit urgent evaluation among HIV-infected and HIV-uninfected pregnant women living in high malaria transmission areas. Because these and prior data suggest that chronicity of placental infection may be associated with poor fetal outcomes, future preventive interventions would ideally target women prior to conception or during early gestation.

## Competing interests

The authors declare that they have no competing interests.

## Authors' contributions

PMN participated in study design, data collection, data analysis and manuscript preparation. HW coordinated data collection in Tororo and participated in data analysis and manuscript preparation. GT designed the molecular marker portion of the study, performed PCR and restriction digestion analysis, and participated in data interpretation. EA participated in coordination of data collection and in interpretation of the data. SW participated in the implementation of the study in Tororo and preliminary data analysis and interpretation. JA participated in study design, provided primary oversight of study activities in Tororo, and reviewed the data and manuscript. DH participated in study design, interpretation of the data and manuscript preparation. PJR conceived the molecular marker portion of the study and assisted in study design, interpretation of the data and manuscript preparation. GD participated in study design and manuscript preparation and oversaw data analysis. TDC implemented the study in Tororo, assisted in the coordination of data collection, was responsible for data management and participated in interpretation of the data and manuscript preparation. DC conceived and designed the study, assisted in analysing and interpretation the data and in drafting the manuscript. All authors read and approved the final manuscript.
